# Heterotopic pregnancy following ovulation induction with clomiphene citrate therapy: A case report

**DOI:** 10.1016/j.crwh.2025.e00757

**Published:** 2025-10-15

**Authors:** Amanda Almeida, Adrianna Zambrano, Elizette Rodriguez, Vidhi Raval, Judith Vinod, Laura Molina, Heather Gabai Hernandez, Adrian Abreu

**Affiliations:** a Nova Southeastern University Dr. Kiran C. Patel College of Osteopathic Medicine, 3200 S University Dr, Fort Lauderdale, FL 33328, United States; b Nova Southeastern University Dr. Kiran C. Patel College of Allopathic Medicine, 3270 S. University Drive, Davie, FL 33328, United States; c Omega Women's Care, LLC, 5695 Coral Ridge Dr, Coral Springs, FL 33076, United States

**Keywords:** Case report, Heterotopic pregnancy, Ectopic pregnancy, Polycystic ovarian syndrome (PCOS), Ovulation-induction

## Abstract

A heterotopic pregnancy is a rare, life-threatening condition in which an intrauterine and ectopic pregnancy occur simultaneously. The incidence of these pregnancies is increasing as the use of assisted reproductive technology and ovulation-inducing agents becomes more common and accessible. A 31-year-old woman with polycystic ovarian syndrome conceived following clomiphene citrate-induced ovulation and was later diagnosed with a heterotopic pregnancy at 10 weeks 2 days of intrauterine gestation and 9 weeks 2 days of ectopic gestation by transvaginal ultrasound following painless vaginal bleeding and rectal pain. The ectopic pregnancy was managed surgically, and the intrauterine pregnancy progressed to a full-term vaginal delivery. This case calls attention to the importance of physicians maintaining a high index of suspicion for heterotopic pregnancy in patients undergoing ovulation induction, despite reassuring imaging and nonspecific symptoms.

## Highlights

• Heterotopic pregnancy occurred after clomiphene in a PCOS patient.
• Normal early ultrasounds masked concurrent ectopic pregnancy.
• Ovulation induction may increase heterotopic pregnancy risk.
• Confirmed intrauterine pregnancy does not rule out ectopic pregnancy.
• Normal β-hCG trajectory cannot exclude heterotopic gestation.

## 1 Introduction

The leading cause of maternal mortality in the first trimester is ectopic pregnancy (EP) [[1], [2], [3], [4], [5]]. An EP occurs when a fertilized egg implants outside the uterine cavity, rendering the pregnancy nonviable and posing significant risk to maternal health [1,2,5,6]. Patients with EP commonly present with lower abdominal pain and vaginal bleeding, with rupture potentially leading to hypovolemic shock, vomiting, and death [1,2,5]. Diagnosis typically involves a positive β-human chorionic gonadotropin (β-hCG) test below expected for gestational age and pelvic ultrasound showing an extrauterine gestational sac [2,3].

This case report presents a patient with a heterotopic pregnancy (HP), a rare, life-threatening condition accounting for 1–3 % of EP cases [5]. An HP is a dizygotic twin pregnancy with one intrauterine and one ectopic implantation, often from abnormal ovulation or asynchronous embryo migration, with an incidence of 1 in 30,000 pregnancies [7]. Although transvaginal ultrasound (TVUS) is highly sensitive (87–99 %) and specific (94–99 %) for EP, early HP diagnosis is challenging, with up to 33 % showing a normal intrauterine pregnancy [8,9]. The prevalence of HP has risen with assisted reproductive technologies such as in-vitro fertilization, intracytoplasmic sperm injection, and intrauterine insemination [7,10,11]. Recent studies have shown that ovulation-inducing agents, such as clomiphene citrate (CC), may also act as a risk factor for HP. [10,12]

CC is first-line pharmacologic therapy for anovulatory infertility, particularly in patients with polycystic ovarian syndrome (PCOS). CC, a selective estrogen receptor modulator, predominantly antagonizes estrogen receptors to stimulate ovulation by altering hypothalamic-pituitary feedback. The typical regimen is 50 mg daily for five days, beginning on day five of the menstrual cycle [13]. CC is well tolerated, with common adverse effects including ovarian enlargement (13.6 %) and vasomotor flushes (10.4 %) [14]. Notably, CC increases the likelihood of multiple gestations, and recent case studies suggest its association with increased risk of HP. [10,12,15]

Given the diagnostic challenges and potential severity of HP, clinicians should maintain a high index of suspicion in patients using ovulation-inducing agents. This case highlights the importance of early recognition and management of HP in patients with PCOS on CC.

## 2 Case Presentation

A 31-year-old woman, gravida 1 para 0, presented to the OB/GYN clinic at 10 weeks 2 days of gestation by last menstrual period (LMP) for a scheduled TVUS due to intermittent, painless vaginal bleeding. Past medical history included PCOS and no prior surgeries. Her menstrual history included irregular cycles when not taking oral contraceptives. She denied a history of sexually transmitted infections, new medications, substance use, or other changes in care. At the time, she was taking 500 mg metformin twice daily and prenatal vitamins. The patient was prescribed 50 mg of CC and took it for two menstrual cycles before conception. At her initial appointment, gestational age was measured at 4 weeks and 1 day by LMP; β-hCG levels rose from 551 mIU/mL to 4281 mIU/mL over five days.

**Fig. 1:**
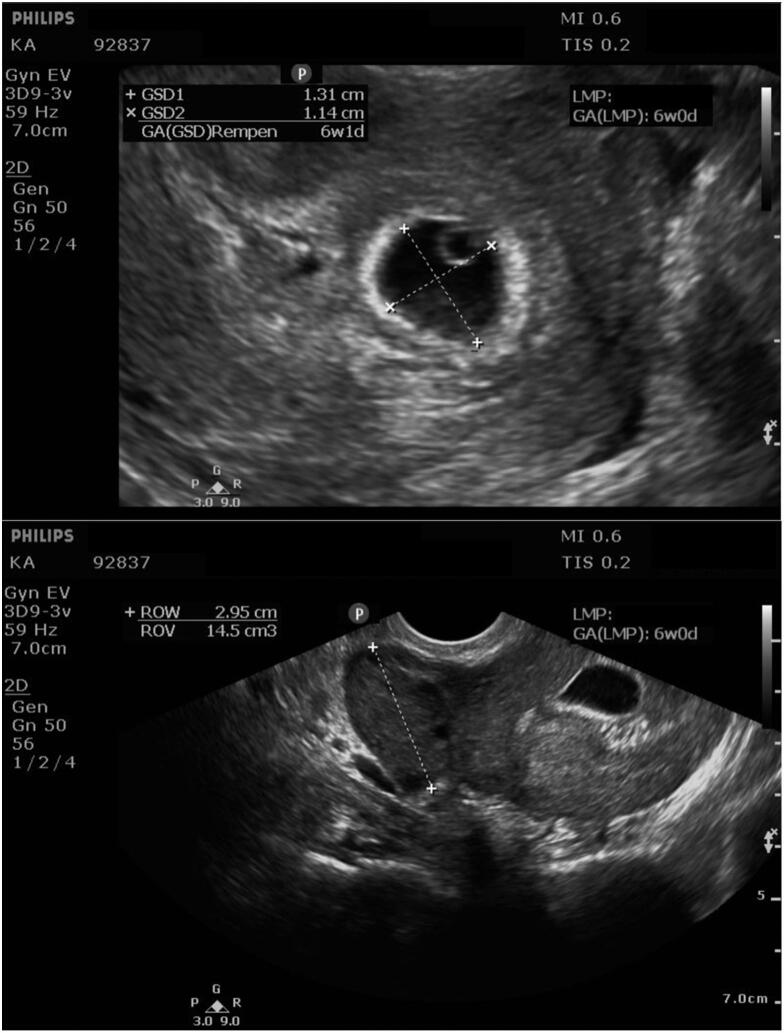
Initial transvaginal ultrasound at 6 weeks of gestation for evaluation of painless vaginal bleeding. Top: Single intrauterine gestational sac without fetal cardiac activity. Bottom: Right ovary within normal limits. *Abbreviations: Gestational Sac Diameter (GSD), Gestational Age (GA), Last Menstrual Period (LMP), Right Ovary Width (ROW), Right Ovary (ROV).*

**Fig. 2:**
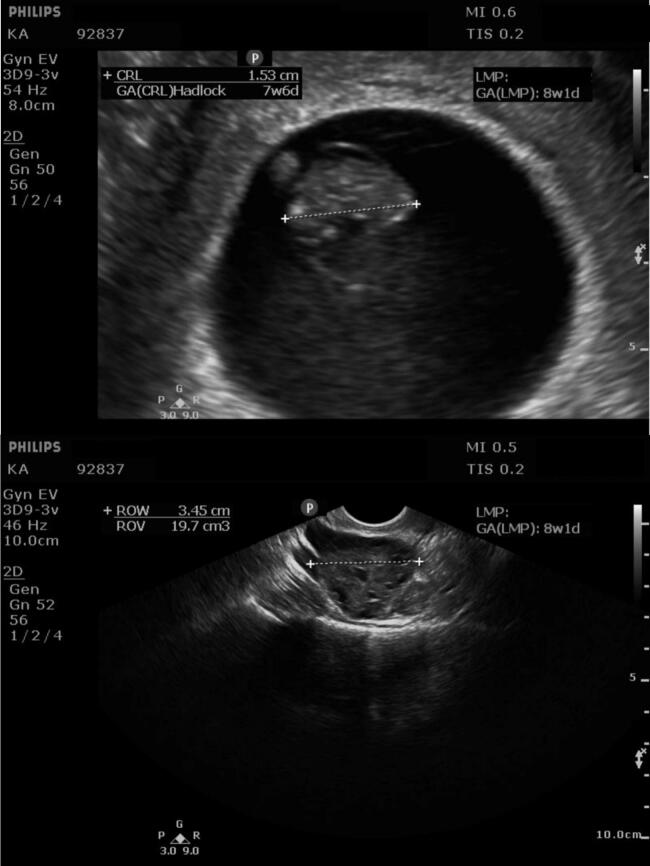
Routine first-trimester transvaginal ultrasound at 8 weeks 1 day of gestation. Top: Single intrauterine gestational sac surrounding the embryo with crown rump length (CRL) of 1.53 cm and adjacent yolk sac. Bottom: Right ovary remains within normal limits.

At 6 weeks of gestation, the patient experienced painless uterine bleeding. Ultrasound evaluation showed a gestational sac without fetal cardiac activity (Fig. 1), normal right ovary, a 1.46 cm simple cyst in the left ovary, and mild free fluid seen in the cul-de-sac. Findings were deemed incidental. Bleeding resolved spontaneously within four days. Two weeks later, ultrasound showed a single live intrauterine pregnancy at 8 weeks 1 day with crown-rump length (CRL) 1.53 cm (Fig. 2). Other findings, including the right ovary, were unchanged.

At 8 weeks 6 days of gestation, the patient reported severe, sudden-onset rectal pain lasting four to six hours. Her physician attributed the pain to constipation, despite no reported bowel habit changes. The pain resolved spontaneously.

At 10 weeks of gestation, the patient noted two days of intermittent, light, painless, brown‑tinged vaginal bleeding. She presented to the clinic at 10 weeks 2 days of gestation, where TVUS showed the previously described intrauterine pregnancy (CRL 3.29 cm) and revealed another pregnancy located in the right adnexa (CRL 2.60 cm) consistent with gestational age of 9 weeks 2 days. Fetal cardiac activity was seen in the intrauterine pregnancy (165 BPM) and questionably detected in the ectopic pregnancy. A large subchorionic hemorrhage and trace blood in the cervix were also visualized. On physical exam, the patient had no tenderness to palpation and no palpable adnexal mass. The patient was notified of the EP and informed that surgery and removal of the EP were the safest treatment option to preserve her life and the life of the intrauterine pregnancy. She was immediately referred to the emergency department, where the EP was confirmed and laparoscopic right salpingectomy was performed the same day.

During the procedure, the right fallopian tube was found to contain an unruptured ectopic fetus that was slowly bleeding bright red blood from the tubal end. The right tube consisted of tan-brown shaggy soft tissue and clotted blood and was successfully removed along with the fragmented fetus without complication.

The surgical pathology final report confirmed the finding of a right fallopian tube with immature chorionic villi, fetal parts, and fetal membranes consistent with products of conception along with adjacent fragments of benign fallopian tube. The patient tolerated the procedure well and recovered without complication, and the intrauterine pregnancy was preserved. The patient had a successful uncomplicated vaginal delivery of the intrauterine pregnancy at 41 weeks 5 days.

## 3 Discussion

This case highlights the diagnostic and management challenges of HP in the setting of CC-induced ovulation. Early diagnosis of HP is particularly challenging due to overlapping symptoms and laboratory findings with normal intrauterine pregnancies, such as nausea and high β-hCG levels. Previous studies have found risk factors for HP include prior tubal damage (ex. from PID, endometriosis, tubal inflammation, mechanical injury), older maternal age, and previous EP. These risk factors parallel the risk factors for EP [16,17]. Additionally, ovulation induction therapies like CC further increase the risk [18].

CC can stimulate polyovulation, often leading to simultaneous intrauterine and ectopic implantations [18]. Additionally, PCOS may itself increase the risk of EP [19]. PCOS causes ovulatory dysfunction and may contribute to EP and HP via altered tubal motility. In normal ovulation, estrogen works to stimulate the fallopian tubes by causing muscular contractions while progesterone relaxes them. Women with PCOS often have elevated estrogen, leading to increased tubal contractility, and, consequently, the risk of EP [20].

Moreover, while serial serum β-hCG measurements are useful in diagnosing EPs, they offer limited utility in distinguishing a HP from singleton or twin intrauterine pregnancy. In viable intrauterine pregnancies, the minimal two-day rise in β-hCG depends on the initial value, about 49 % when below 1500 mIU/mL [17]. In this patient, β-hCG increased from 551 mIU/mL at 4 weeks 1 day to 4281 mIU/mL at 4 weeks 6 days, an eightfold rise consistent with a viable intrauterine pregnancy. This appropriate trajectory masked the concurrent adnexal gestation, making it difficult to suspect on laboratory evaluation alone. In addition, symptoms such as painless vaginal bleeding and rectal pain, as seen in this patient, are non-specific and may overlap with normal early pregnancy findings. Nonetheless, the patient's doctor later attributed the findings of rectal pain to the growing EP exerting pressure on the rectum and surrounding nerves.

**Fig. 3:**
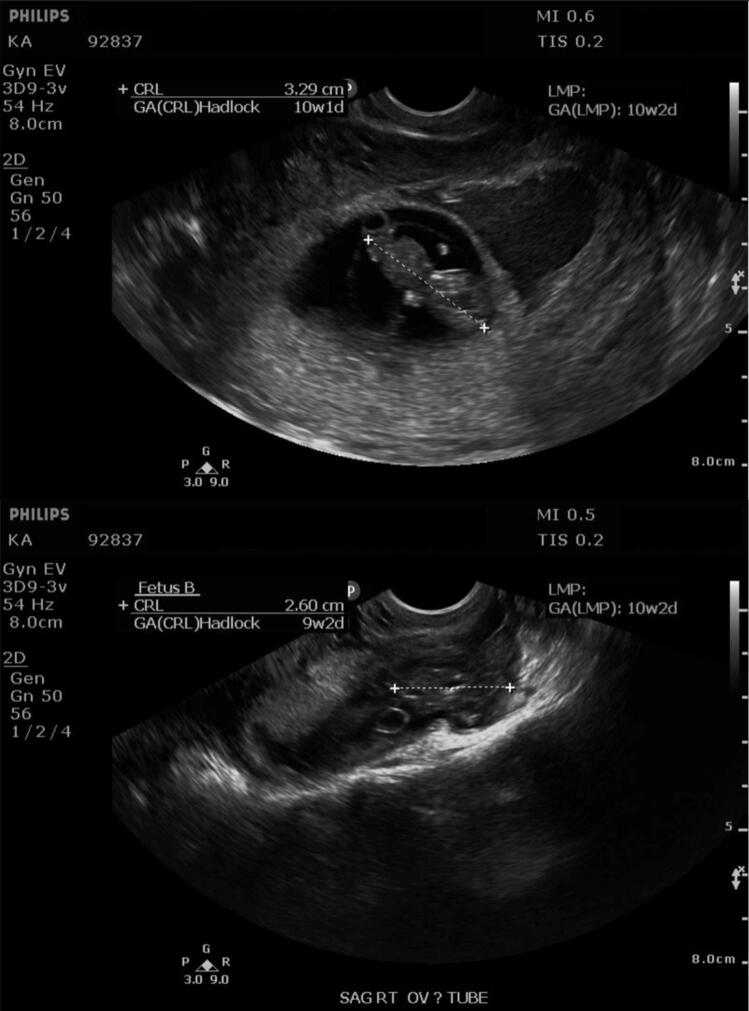
Transvaginal ultrasound at 10 weeks 2 days of gestation after recurrence of painless bleeding. Top: Single intrauterine pregnancy with CRL of 3.29 cm and adjacent yolk sac. Bottom: Right adnexa displays a second gestational sac, “Fetus B", with CRL of 2.60 cm corresponding to 9 weeks 2 days of gestation by Hadlock dating, consistent with an ectopic pregnancy.

Lastly, the absence of a visualized ectopic pregnancy on TVUS at 8 weeks of gestation is highly unusual. By 5 to 6 weeks, a gestational sac or adnexal mass is usually appreciated on TVUS with high sensitivity and specificity, regardless of pregnancy location [3,8]. In this case, the EP remained undetected at 8 weeks despite appropriate technique, an experienced sonographer, and a fully functional machine. All three ultrasound scans were performed in a clinical setting by the same technician, and multiple additional views of the uterus and adnexa were obtained at each visit. For consistency, representative images of the uterus and right ovary are presented here (Fig. 1, Fig. 2, Fig. 3); however, none demonstrated the adnexal pregnancy until 10 weeks. The reassuring finding of a viable intrauterine pregnancy, coupled with the absence of any adnexal abnormality on transvaginal ultrasound at 8 weeks 1 day (ectopic fetus would be 7 weeks 1 day gestation by LMP), lowered clinical suspicion and contributed to the delayed recognition of the heterotopic gestation.

In summary, this case highlights the inherent challenges of diagnosing HP in patients undergoing ovulation-induction therapy. Early and thorough evaluation, including careful interpretation of ultrasound scans and β-hCG levels, is paramount. In this patient, the presence of the EP was not noted on her first two ultrasound examinations. Prompt recognition and timely intervention not only mitigate maternal morbidity and mortality but also help preserve the viability of intrauterine pregnancy.

Despite the present standard diagnostic protocols, the overlapping clinical presentation complicated early identification of the EP in this patient. This underscores the critical need for a high index of suspicion for HP in patients receiving ovulation-inducing therapies, especially in those with additional risk factors such as PCOS. As seen in the present case, relying solely on present diagnostic modalities may be insufficient, and to detect EPs earlier a proper protocol must be established involving the integration of imaging and risk factors into the diagnostic process. Further research is warranted to refine screening guidelines to ultimately improve early detection strategies, reduce maternal morbidity, and optimize fetal outcomes.

## Contributors

Amanda Almeida contributed to patient care, the conception of the case report and acquiring the data.

Adrianna Zambrano contributed to acquiring and interpreting the data, conducting the literature review and revising the article critically for important intellectual content.

Elizette Rodriguez contributed to acquiring and interpreting the data and drafting the manuscript.

Vidhi Raval contributed to acquiring and interpreting the data and undertaking the literature review.

Judith Vinod contributed to acquiring the data and drafting the manuscript and revising the article critically for important intellectual content.

Laura Molina contributed to patient care and revising the manuscript for important intellectual content.

Heather Gabai Hernandez contributed to patient care and revising the manuscript for important intellectual content.

Adrian Abreu contributed to revising the manuscript for important intellectual content.

All authors approved the final submitted manuscript.

## Patient consent

Written informed consent was obtained from the patient for publication of this case report and accompanying images.

## Provenance and peer review

This article was not commissioned and was peer reviewed.

## Funding

No funding from an external source supported the publication of this case report.

## Declaration of competing interest

The authors declare that they have no competing interest regarding the publication of this case report.
